# Combined effects of leaks, respiratory system properties and upper airway patency on the performance of home ventilators: a bench study

**DOI:** 10.1186/s12890-017-0487-2

**Published:** 2017-11-21

**Authors:** Kaixian Zhu, Claudio Rabec, Jésus Gonzalez-Bermejo, Sébastien Hardy, Sami Aouf, Pierre Escourrou, Gabriel Roisman

**Affiliations:** 1 Centre Explor, Air Liquide Healthcare, 28 rue d’Arcueil, 94250 Gentilly, France; 2 Service de Pneumologie et Soins Intensifs Respiratoires, Centre Hospitalier Universitaire Dijon Bourgogne, 14 rue Paul Gaffarel, F-21079 Dijon, France; 3 Sorbonne Universités, UPMC Univ Paris 6, INSERM, UMRS1158 Neurophysiologie Respiratoire Expérimentale et Clinique, Paris, France; 4 Service de Pneumologie et Réanimation Médicale (Département “R3S”), AP-HP, Groupe Hospitalier Pitié-Salpêtrière Charles Foix, F-75013 Paris, France; 5 Service des Explorations Fonctionnelles Multidisciplinaires, AP-HP, Hôpital Antoine-Béclère, 157 rue de la Porte de Trivaux, 92140 Clamart, France

**Keywords:** Non-invasive ventilation, Patient-ventilator asynchrony, Unintentional leak, Upper airway patency

## Abstract

**Background:**

Combined effects of leaks, mechanical property of respiratory system and upper airway (UA) patency on patient-ventilator synchrony (PVA) and the level of clinically “tolerable” leaks are not well established in home ventilators.

**Methods:**

We comparatively assessed on a bench model, the highest leak level tolerated without inducing significant asynchrony (“critical leak”) in three home ventilators (Astral 150, Trilogy 100 and Vivo 60; noted as A150, T100 and V60 respectively) subjected to three simulated diseased respiratory conditions: chronic obstructive pulmonary disease (COPD), obesity hypoventilation (OHS) and neuromuscular disorders (NMD), with both open and closed UA. Also, total leak values in the device reports were compared to the bench-measured values.

**Results:**

With open UA, all ventilators were able to avoid asynchrony up to a 30 L/min leak and even to 55 L/min in some cases. UA closure and respiratory diseases especially OHS influenced PVA. With closed UA, the critical leak of A150 and T100 remained higher than 55 L/min in COPD and OHS, while for V60 decreased to 41 and 33 L/min respectively. In NMD with closed UA, only T100 reached a high critical leak of 69 L/min. Besides, inspiratory trigger sensitivity change was often necessary to avoid PVA.

**Conclusions:**

Home ventilators were able to avoid PVA in high-level leak conditions. However, asynchrony appeared in cases of abnormal mechanical properties of respiratory system or closed UA. In case of closed UA, the EPAP should be adjusted prior to the inspiratory trigger.

**Trial registration:**

Not applicable.

**Electronic supplementary material:**

The online version of this article (10.1186/s12890-017-0487-2) contains supplementary material, which is available to authorized users.

## Background

Home non-invasive ventilation (NIV) is a well-established treatment for patients with chronic hypercapnic respiratory failure so that the number of patients equipped with NIV devices has been steadily increasing all over the world [1–3]. To prevent the progression of sleep time toward daytime hypercapnia and further severe respiratory failure, home NIV is predominantly applied during sleep time.

There are two specific characteristics of NIV that affect the ventilation effectiveness, particularly during sleep. First, because NIV is delivered via a nasal or full-face mask, unintentional leak is a commonly found drawback as a consequence of the non-hermetic nature of the system [4–6]. Leakage may be absent or minimal when the patient is awake but often worsen during sleep as a result of the loss of voluntary control and decreased muscle tone. Second, under NIV, intermittent obstruction of the upper airway (UA) is commonly observed during sleep in subjects with increased UA collapsibility. In chronic NIV, both obstructive breathing events and unintentional leaks are known to induce the occurrence of patient-ventilator asynchronies (PVA), which may reduce both ventilator efficacy and sleep quality [7–9] and are associated with decreased patient survival [10, 11].

Adjustable inspiratory triggering which is more sensitive than classical flow triggering is supposed to reduce PVA. Moreover, with latest generation blowers, very high-level leaks can be compensated. Nevertheless, the level of leaks that can be tolerated by the NIV ventilators for maintaining patient-ventilator synchronization is unknown.

The goal of this study was to comparatively assess in a bench model, the maximal leak level tolerated without inducing significant asynchrony (“critical leak”) in ventilators subjected to three diseased respiratory conditions: chronic obstructive pulmonary disease (COPD), obesity hypoventilation syndrome (OHS) and neuromuscular disorders (NMD). Each disease was simulated with both open and closed UAs.

## Methods

### Bench model

**Fig. 1:**
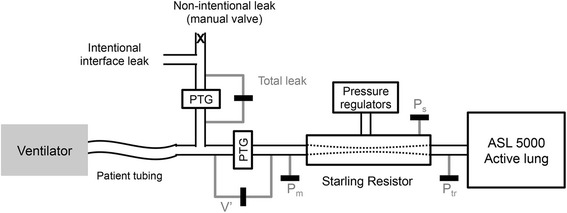
The principle of the bench model. PTG: pneumotachograph; V′: mask flow; Pm: Mask pressure; Ptr: tracheal pressure; Ps: pressure in the Starling resistor

Evaluations were carried out on a bench set-up that has been modified from a previously described respiratory model [12]. The bench model consisted of an active lung ASL5000 (IngMar Medical, Pittsburgh, USA) and a Starling resistor. This system allows simulating physiological and pathological conditions of the respiratory system, by varying respiratory mechanics, inspiratory effort as well as UA patency. The modified bench model (Fig. 1) included a variable-opening valve located downstream to the calibrated intentional nasal leak port (24 L/min at 10 cmH2O) to simulate different levels of unintentional leak. The total leak was continuously measured by a pneumotachograph with digital display and analog output (Model 4040 Flowmeter, TSI Inc., MN, USA), which is linear in the studied leak flow ranges. A manometer (MecoSmart D-06, Mesureur, Chilly-Mazarin, France) was added to measure the pressure downstream to the Starling resistor (Tracheal pressure, Ptr).

### Simulated diseased respiratory conditions and active lung model settings

Three diseased respiratory conditions were simulated: COPD, OHS and NMD. The patient’s inspiratory effort was simulated by specifying a “muscular” pressure (Pmus) (equivalent to pleural pressure) in the ASL5000 [13]. In each breathing cycle, inspiration and expiration correspond to the contraction and relaxation phases of the inspiratory muscles, during which the Pmus can be represented by two exponential functions, respectively. These functions are characterized by the patient’s breathing rate, the drop in muscular pressure at 100 ms (Pmus 0.1) reflecting patient’s ventilatory drive [14] and the maximum Pmus decrease (Pmax) during the breathing cycle [15]. The values of inspiratory effort settings in the ASL5000 for the three simulated diseases were chosen according to published clinical values [16–20]. The ASL5000 settings corresponding to the simulated respiratory conditions are shown in Additional file 1 [21, 22].

### Simulated upper airways patency patterns

For each diseased condition, we simulated open and closed UA by applying external pressures in the Starling resistor (Ps) of −8 and +8 cmH2O respectively. The simulated airflow, the corresponding inspiratory muscular pressure and tidal volume of the three diseased conditions with open and closed UA are shown in Additional file 1.

### Tested devices and ventilatory settings

Three life support home ventilators were evaluated in the study: Astral™ 150 (Resmed, NorthRyde, Australia; software version SX544-0301; denoted as A150), Trilogy™ 100 (Philips Respironics, Monroeville, USA; software version 14.1.02; denoted as T100) and Vivo™ 60 (Breas, Mölnlycke, Sweden; firmware version 3.05; denoted as V60). These devices were able to provide pressure-targeted ventilation with single-limb circuit configuration including an intentional leak. Because inspiratory trigger sensitivity was graduated in different units in each device, these settings were first comparatively evaluated with ASL5000 to establish equivalent sensitivities among devices. The details of methods and results are provided in Additional file 2.

Besides, other ventilatory settings such as rise time and I:E cycling were also comparatively evaluated and the corresponding comparisons are available in Additional file 2. Table 1 shows the chosen ventilatory settings for the current study.

**Table 1:** Applied ventilator settings

	A150	T100	V60
Mode	ST	ST	Support
EPAP-IPAP (cmH_2_O)	5-15	5-15	5-15
Backup respiratory rate (bpm)	7	7	7
I:E cycling	High	40%	5 (a.u.)
Rising time	200 (ms)	1 (a.u.)	2 (a.u.)
Ti (s)	0.8-1.5	1.5	0.8-1.5

*ST* spontaneous triggered pressure support mode with backup rate, *EPAP* expiatory positive airway pressure, *IPAP* inspiratory positive airway pressure, *Ti* inspiratory time, *bpm* breaths per minute. *A150* Astral™ 150, *T100* Trilogy™ 100, *V60* Vivo™ 60, *a.u.* arbitrary unit

### Patient-ventilator asynchrony: ineffective efforts and autotriggering

PVA is defined as the mismatching between neural and mechanical inspiratory time [23–25]. Phase asynchronies, characterized by a mismatch between the start or the end of the patient’s and the ventilator’s inspiratory time are the most common asynchronies. They may occur at two periods: during inspiratory triggering, in situations in which there is a mismatch between patient inspiratory effort and ventilator triggering (i.e.: ineffective inspiratory effort, double triggering or autotriggering) or during cycling from inspiration to expiration, when ventilator cycling does not coincide with the end of patient effort (i.e.: premature or delayed cycling) [26].

**Fig. 2:**
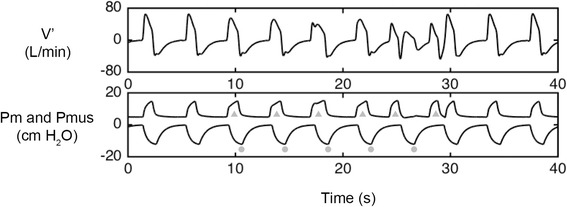
Example of patient-ventilator asynchrony events. V′: mask airflow; Pm: mask pressure; Pmus: inspiratory muscular pressure. An ineffective effort was defined as a simulated inspiratory effort that was not followed by an assisted pressure support in Pm (dot); An autotriggered cycle was defined as a non-backup ventilatory cycle that was delivered by the ventilator during the expiratory phase without a prior Pmus decrease (triangle)

In the current study, PVA during the triggering phase was characterized as a mismatch between the simulated inspiratory effort and the occurrence of the ventilator assisted cycle, which is reflected by whether or not a ventilatory cycle occurs following a decrease in Pmus [23–25]. Thus, an ineffective effort was defined as a simulated inspiratory effort that was not followed by an assisted cycle (i.e., delay longer than the inspiratory phase) and an autotriggered cycle as a non-backup cycle that was delivered by the ventilator during the expiratory phase without a prior Pmus decrease (Fig. 2). As the backup rate was set at 7 bpm, an autotriggered cycle could occur within 8.6 s after the beginning of the previous cycle.

The asynchrony level (AL) was defined as the ratio of the number of asynchrony events over the sum of the numbers of ventilatory cycles and ineffective efforts [26]. Therefore, the PVA included both ineffective efforts and autotriggered cycles.

### Protocol

The ventilator was connected to the calibrated leak port built in the bench model through a 1.8-m long and 22-mm diameter tubing. The principal endpoint was to find for each ventilator the “critical leak” level at which the asynchrony appeared in COPD, OHS and NMD conditions with either open or closed UAs. The AL was calculated for each 5-min period at different leak levels. The “critical leak” was defined as the median value of the highest total leak level (intentional + unintentional leaks) during 5 min at which the AL remained lower than 25%.

**Fig. 3:**
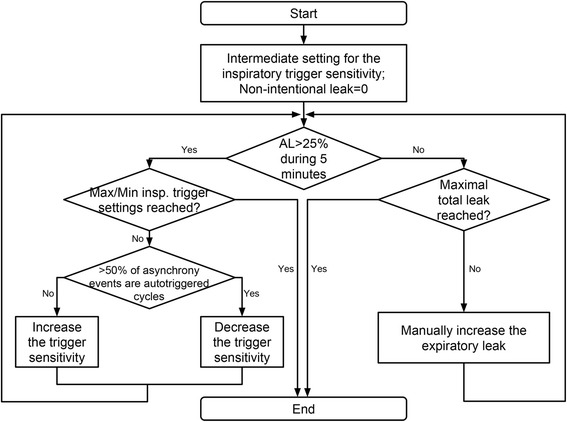
The protocol for finding the « critical leak » of ventilators. AL: asynchrony level (ratio of the number of asynchrony events over the sum of the numbers of ventilatory cycles and ineffective efforts)

Figure 3 shows the algorithm used in the protocol for finding the “critical leak”. Each test started with the intermediate inspiratory trigger setting of ventilator (“Medium” for A150, “5” for T100 and V60) since these settings were graduated in different units in each device and did not correspond to each other (Additional file 2). The total leak started with the minimum level, i.e., intentional leak with closed valve that simulated the unintentional leak (Fig. 3). At the end of each 5-min period, if the AL was lower than 25%, the total leak would be manually increased in a stepwise manner by regulating the total end-expiratory leak in the following order: 30, 40, 45, 50 and 55 L/min; otherwise, the last leak level was maintained while the inspiratory trigger was adjusted in a stepwise manner to decrease the PVA events as follows: increase the trigger sensitivity if predominant events (> 50%) were ineffective efforts, or decrease the trigger sensitivity if predominant events (> 50%) were autotriggered cycles. This procedure was repeated until the critical leak was reached. An “effective adjustment” was defined as a manual adjustment of trigger sensitivity that decreased AL below 25%. In each test, the inspiratory trigger settings that corresponded to the critical leak were noted and the number of repetitions necessary to obtain the final result was noted. In addition, leak values were compared between device reports and that measured on the bench. Details are presented in Additional file 3.

### Data analysis

The following data were recorded with a sampling frequency of 20 Hz: mask airflow (V′), mask pressure (Pm), tracheal pressure (Ptr), pressure in the Starling resistor (Ps) and total leak. The muscular pressure (Pmus) was exported from the ASL5000 software (IngMar Medical, Pittsburgh, USA) and was synchronized to the bench-recorded data for further analyses. For each 5-min period during which the inspiratory trigger and total leak level were unchanged, the number of autotriggered cycles and ineffective efforts were calculated by analyzing the Pm and Pmus curves. As leak flow follows the airway pressure, the distribution of leak values relates to that of the airway pressure. High leaks occurred at inspiratory phases when pressure support was applied; during the rest of time, a constant lower leak corresponding to the expiratory pressure persisted (i.e., the expiratory leak). The leak values during ventilatory cycles were thus not normally distributed. Consequently, the “critical leak” was presented as median (range, min-max values) of the 5-min period. Comparisons of “critical leaks” between devices for each diseased respiratory and UA conditions were performed using Kruskal-Wallis tests. Statistical analyses were performed with MedCalc (MedCalc Software, Mariakerke, Belgium).

## Results

**Fig. 4:**
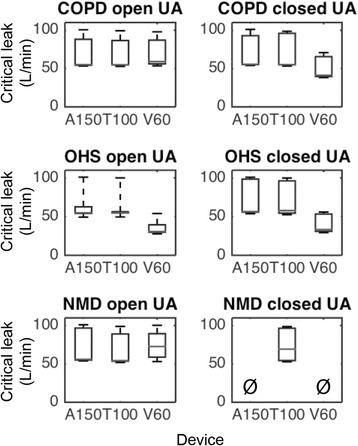
“Critical leak” of the ventilators in three respiratory conditions with open and closed upper airway. COPD: chronic obstructive pulmonary disease; OHS: obesity hypoventilation syndrome; NMD: neuromuscular disorder; UA: upper airway. A150: Astral 150; T100: Trilogy 100; V60: Vivo 60. Box plots show median, interquartile range (25-75th percentiles), minimum and maximum values of the highest leak level during 5 min at which the asynchrony index remained lower than 25%. In some conditions, the median and the 25th percentile are similar (e.g., A150 and T100 in COPD with open UA). Ø: no data

Figure 4 and Additional file 4 show, respectively, the “critical leak” and the corresponding inspiratory trigger settings of the three home ventilators according to the simulated respiratory diseases.

**Fig. 5:**
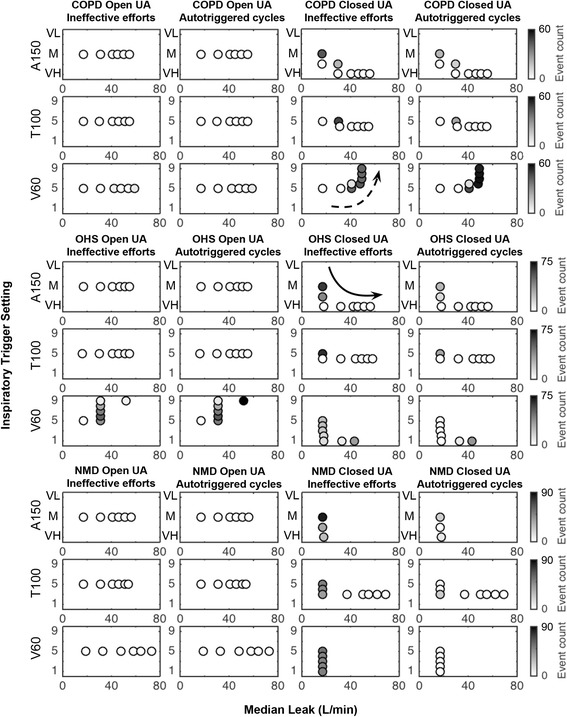
Evolutions of patient-ventilator inspiratory asynchrony events of the three ventilators in relationship to leak level and trigger sensitivity during the tests. This figure illustrates the evolutions of the amount of ineffective efforts and autotriggered cycles with various leak levels and inspiratory trigger settings in the three simulated respiratory conditions with open and closed upper airways (UA) during each test according to the protocol in Fig. 3. Each point represents a 5-min period and grey intensity of the points reflects the amount of asynchrony events. The number of asynchrony events could be decreased at a certain total leak level by changing the inspiratory trigger sensitivity of the ventilator. Also, according to the protocol, the total leak level was manually increased in a monotone way by regulating the end-expiratory leak (following the arrow for each situation). The median total leak values are shown in this figure. As an example (line 6, columns 1 and 2), in OHS with open UA, the inspiratory trigger sensitivity of V60 was initially set at 5 and the median total leak was equal to 17 L/min (intentional leak only). Since no asynchrony events were found during the first 5-min test, according to the protocol (Fig. 3), the total leak was manually increased and the median total leak value reached 31 L/min. At this leak level, the number of ineffective efforts and autotriggered cycles increased to 37 and 44 respectively and Asynchrony Level (AL) reached ≥25%. As autotriggering was the predominant asynchrony event, the trigger sensitivity was decreased in a stepwise manner till the AL became <25%. This was achieved when inspiratory trigger was set at 9, and the corresponding number of ineffective efforts and autotriggered cycles were decreased to 6 and 9 respectively (AL = 18%). According to the protocol, the total leak then immediately increased to the next total leak level and the median total leak reached 52 L/min. In this condition, the AL became 53% (5 ineffective efforts and 74 autotriggered cycles). As the most insensitive trigger (=9) was reached for this device, the precedent median total leak (31 L/min) was thus considered as the “critical leak” for V60 in OHS with open UA condition. Solid and dotted arrows indicate stepwise increase and decrease of inspiratory trigger sensitivity, respectively. COPD: chronic obstructive pulmonary disease; OHS: obesity hypoventilation syndrome; NMD: neuromuscular disorder; AI: asynchrony index; A150: Astral 150; T100: Trilogy 100; V60: Vivo 60. VL: very low, M: medium, VH: very high

With open UAs, all ventilators were able to avoid PVA in any lung conditions with critical leaks up to 31 L/min, and even up to 55 L/min for some devices (Fig. 4). In OHS situation, the inspiratory trigger sensitivity of V60 was manually adjusted 4 times to most insensitive (setting 9) while autotriggered cycles still persisted when leak exceeded 31 L/min (Fig. 5). Under other conditions, all ventilators were able to avoid both ineffective efforts and autotriggered cycles regardless the level of leak (Fig. 5, also see Additional file 4).

With closed UAs the results were heterogeneous between the simulated respiratory conditions and between devices. In COPD and OHS conditions, all ventilators were able to avoid PVA although the median critical leak was lower for V60 (41 and 33 L/min for COPD and OHS, respectively) compared with A150 and T100 (higher than 55 L/min each; see Figs. 4 and 5). In NMD condition, only T100 reached a critical leak of 69 L/min (Fig. 4) while the other two ventilators failed to detect the inspiratory efforts even in the case of total leaks as low as 17 L/min with the most sensitive inspiratory trigger settings (“very high” for A150 and “1” for V60; see Figs. 4 and 5). An example of the patient-ventilator asynchronies with closed UAs is given in Additional file 5. With closed UAs, the inspiratory trigger was changed 22 times in total in 9 PVA situations (all ventilators in all lung conditions), in which 5 PVA situations were alleviated (AL < 25%) in the end after regulating the trigger sensitivity.

Results of comparison of leaks between device reports and bench-measured values are presented in Additional file 3.

## Discussion

The goal of the current study was to evaluate the combined effects of leaks, mechanical properties of the respiratory system and UA patency on patient-ventilator synchrony during the triggering phase. We comparatively assessed on a previously reported bench model [12], the highest leak level tolerated without inducing significant PVA (“critical leak”) in three home ventilators subjected to three simulated diseased respiratory conditions such as COPD, OHS and NMD. Each disease was simulated with both open and closed UAs. In addition, total leak values in the device reports were compared to the bench-measured values. The main findings were as follows: 1) with open UAs, all the ventilators were able to avoid PVA up to a 31 L/min leak and even up to 55 L/min in some cases. 2) UA closure and respiratory diseases, especially OHS, dramatically influenced PVA. With closed UAs, the critical leaks of A150 and T100 remained higher than 55 L/min in COPD and OHS, while for V60 these values decreased to 41 and 33 L/min, respectively. In NMD with closed UAs, only T100 reached a high critical leak of 69 L/min. 3) Inspiratory trigger sensitivity change is often necessary to avoid PVA.

Since leak is the first cause of failure of NIV, inspiratory trigger performance of ventilators need to be tested in presence of leak. We confirm here the improvement of the inspiratory triggers in new ventilators: without UAs closure, all the ventilators were able to avoid PVA up to 30 L/min leaks, and even up to 55 L/min sometimes. These new flow-based triggering algorithms are more sensitive than classical flow triggering, and allow adjusting trigger sensitivity in presence of leak and, in this way, help to reduce ineffective efforts and auto-cycling. Using an “NIV algorithm” in ICU ventilators has been shown to significantly decrease the impact of leaks on the occurrence of patient-ventilator trigger asynchrony in some clinical cases [23]. But to our knowledge, our study is the first to systematically and comparatively evaluate the effect of unintentional leaks on triggering function in different ventilators using sophisticated flow-based trigger algorithms.

It has been stated that an unintentional leak lower than 24 L/min is clinically tolerable in most cases [27]. Our study shows that the adverse effects of leaks during the triggering phase vary according not only to the absolute leak level but also to the mechanical properties of the lung and UA patency. The type of ventilator also affects the negative effect of leaks on the occurrence of PVA. We show that the threshold value of clinically “tolerable” leaks must be determined for each ventilator and that these thresholds differ between different devices and/or clinical situations. We also show that, without UA obstruction, all ventilators performed well with leaks lower than 30 L/min which are clinically tolerable [27].

Clinical situations may also lead to PVA: UA obstruction and respiratory diseases. Most studies evaluating ventilator performance tested them by applying different respiratory mechanics conditions and leaks levels [28–31]. The originality of our lung model is the use of a Starling resistor simulating UA closure on a bench model that takes into account the pressure responses of tested devices in a “closed loop” setting (i.e. with a direct interaction of the ventilator to the bench condition). To our knowledge, no study has evaluated so far the influence of varying UA resistance on leak-induced patient-ventilator trigger asynchronies.

Intermittent obstruction of UA during sleep is common under NIV and may be related to obstructive events at the oropharyngeal level (obstructive sleep apnea-hypopnea) because of insufficient expiratory positive airway pressure (EPAP) during sleep, in subjects with increased UA collapsibility. Currently, obstructive sleep apnea-hypopnea may be observed in all patients receiving NIV, in particular those with OHS. As assisted ventilator cycles are triggered either by the pressure drop in the proximal airway or by the detection of flow variation in the presence of a continuous flow washing out the circuit during expiration, this reduction in UA patency may reduce the capacity of the ventilator to detect patient inspiratory effort. Therefore, the EPAP should be increased prior to any changes in inspiratory trigger sensitivity when obstructive respiratory events occur, especially for low breathing efforts such as the case of neuromuscular diseases.

Finally, adjustable inspiratory trigger is an option presently available in most home ventilators and our results suggest that manual adjustment of the inspiratory trigger sensitivity allows a decrease of PVA. After verifying leak and EPAP levels, the inspiratory trigger sensitivity can be adjusted depending on the type of asynchrony events as described in our protocol (Fig. 3). However, in routine, this can be difficult as patient’s conditions change daily. An automatic adaptation of the trigger sensitivity, taking into account the presence of leaks but also of PVA could be useful in the future.

Currently, home ventilators allow leaks to be estimated, but the performance of their algorithms is variable [32]. Also, intentional leaks are variable and proportional to the level of pressure. They differ between interfaces, and are included in the estimation of leaks in most algorithms [33]. Our study confirms data of Contal et al. concerning the differences in the accuracy of several devices in estimating unintentional leaks. In our study, only one device (T100) accurately estimated leak values at all tested levels as compared with bench-measured values. Moreover, in one of the devices (V60) this bias enlarged as the leak level increased. Thus, our study suggests that detecting and knowing accurately the magnitude of unintentional leaks and particularly their impact on the quality of ventilation is of major importance when monitoring NIV. Also, the methods of calculating leaks applied by ventilators remain to be standardized, especially those concerning the timing within a breathing cycle at which leaks are measured.

Some limitations may be made with regard to our study. First, we used a respiratory bench model. Even if advantages of this approach are evident (standardization of mechanical characteristics, repeatability, ability to study a broad range of controlled situations), this is certainly a simplified representation of a patient’s complex inspiratory effort profile and pulmonary mechanics. Next, in the clinical setting this profile may vary over time. As an example, long term NIV is mainly applied during sleep, a physiological state characterized by variations in ventilatory drive, respiratory mechanics, UA patency and respiratory muscle recruitment during different sleep stages and changes in body position. Last, there are inherent limitations to bench testing per se: we applied a step-by-step leak increment. Indeed, the model does not integrate the inherent variability of unintentional leaks when the amount of leaks may rapidly vary in few cycles or even during the same cycle. Taking into account these limitations, there is a body of literature that suggests that bench testing is, however, an important and relevant component of the assessment of home ventilators. We evaluated three ventilators and applied our model to three simulated diseased respiratory conditions that may not be representative neither of the broad spectrum of available home ventilators nor reflecting the large range of clinical situations.

## Conclusions

In conclusion, we demonstrated that performances of modern home ventilators are able to avoid PVA at much higher level of leaks than previously published. Unfortunately, in case of abnormal mechanical properties of the respiratory system or closed UA this function is strongly compromised with very heterogeneous results and manual changes in trigger sensitivity are often necessary to avoid PVA. Difference in coping with leaks and in the capacity of estimating leaks may account for between-device differences in effectiveness of NIV. As detecting and knowing unintentional leaks are of major importance when monitoring NIV, physicians must be aware of these discrepancies and must be able to set the sensitivity of the inspiratory triggers although this is usually difficult. Besides, in case of closed UA, the first setting to adjust should be the EPAP rather than the inspiratory trigger. Automatic detection of leaks and adaptation of triggering sensitivity by the devices is desirable, but further efforts are needed to improve leak estimation and ventilator performance during leaks and to clarify how leaks must be detected and understood: when are they “acceptable” and at what point do they become a problem.

## Additional files

Additional file 1: Active lung model settings and simulated breathing patterns. Details of simulation of diseased breathing patterns. (PDF 144 kb)

Additional file 2: Study of equivalent ventilatory settings. Following ventilatory settings were analyzed: inspiratory trigger sensitivity, pressure rise time and I:E cycling. (PDF 265 kb)

Additional file 3: Comparison of leaks between device reports and bench-measured values. Protocol and results of comparing the leak values between device reports and bench measures. (PDF 232 kb)

Additional file 4: Inspiratory trigger settings corresponding to “critical leak” values. Table of the inspiratory trigger settings of the three ventilators corresponding to the “critical leaks” according to the protocol in Fig. 3. (PDF 80 kb)

Additional file 5: Example of patient-ventilator asynchronies with closed upper airways. Detailed curves of airway flow, pressures and leaks illustrating observed patient-ventilator asynchronies. (PDF 5414 kb)

## Abbreviations

AL: Asynchrony level
COPD: Chronic obstructive pulmonary disease
EPAP: Expiratory positive airway pressure
NIV: Non-invasive ventilation
NMD: Neuromuscular disorders
OHS: Obesity hypoventilation
Pmax: Maximum Pmus decrease
Pmus: Muscular pressure
Ps: Pressure in the Starling resistor
Ptr: Tracheal pressure
PVA: Patient-Ventilator Asynchrony
UA: Upper Airway
