# Supplementary material for: Combined effects of leaks, respiratory system properties and upper airway patency on the performance of home ventilators: a bench study
Source: BMC Pulm Med. 2017 Nov 21;17:145. doi: 10.1186/s12890-017-0487-2 (PMC5697337; doi:10.1186/s12890-017-0487-2)
Supplement: Supplementary file 1 — Active lung model settings and simulated breathing patterns. Details of simulation of diseased breathing patterns. (PDF 144 kb) [file 12890_2017_487_MOESM1_ESM.pdf]

Combined effects of leaks, respiratory system properties and upper airway patency on the performance of home ventilators: A bench study

Kaixian Zhu, Claudio Rabec, Jésus Gonzalez-Bermejo, Sébastien Hardy, Sami Aouf, Pierre Escourrou and Gabriel Roisman

Additional File 1: Active lung model settings and simulated breathing patterns

Table S1-1. The settings of ASL5000 corresponding to the simulated respiratory diseases

		COPD	OHS	NMD
Resistance ($cmH_2O·L·s^{-1}$)*	Inspiration	20	3	3
	Expiration	25		
Compliance ($ml·cmH_2O^{-1}$)**		50	30	60
P_{mus} at 0.1 second (cmH_2O)		3	3	1
P_{max} (cmH_2O)		13	12	4.3
Breathing rate (bpm)		12	15	20
T_I/T_E		1/2	1/2	1/1.6

COPD: chronic obstructive pulmonary disease, OHS: obesity hypoventilation syndrome, NMD: neuromuscular disorder. Pmus: inspiratory muscular pressure; Pmax: maximum pressure drop during breathing cycle. bpm: breath per minute; T_I: inspiratory time; T_E: expiratory time.

*Values of resistance of 5, 10, 20, and 50 cmH2O/L/s characterize absent, moderate, severe, and extreme obstruction, respectively [Olivieri 2011].

**Values of compliance of 100, 50, and 25 mL/cmH2O characterize absent, mild, and severe restriction [Olivieri 2011]. Of note, since severe COPD is usually associated with hyperinflation and the end-expiratory tidal volume is shifted forward the upper flat portion of the V-P relationship in these patients.

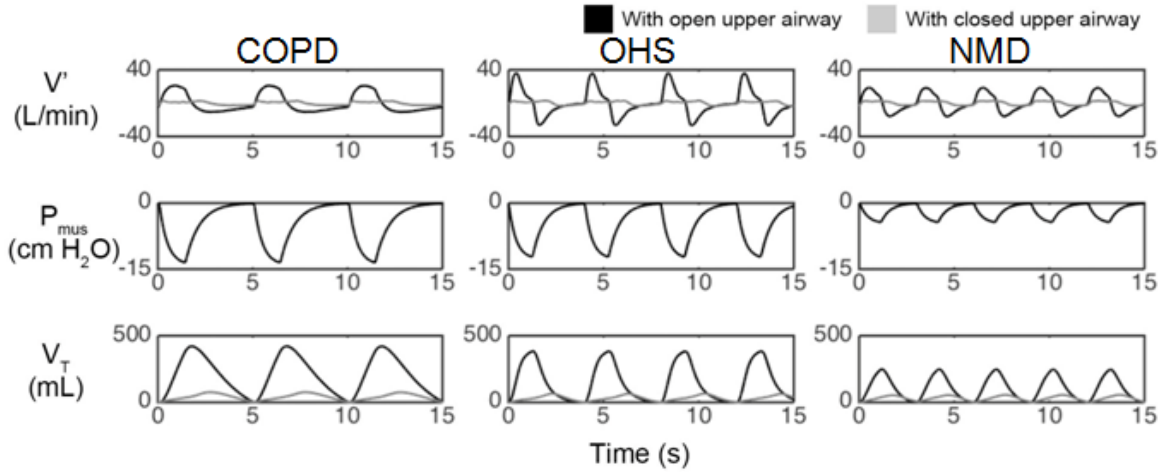

Figure S1-1: The simulated airflow (V'), the corresponding inspiratory muscular pressure (Pmus) and the resulting tidal volume (V_T) of the three lung diseases with open and closed upper airways. COPD: chronic obstructive pulmonary disease; OHS: obesity hypoventilation syndrome; NMD: neuromuscular disorder.

Olivieri C, Costa R, Conti G, Navalesi P. Bench studies evaluating devices for non-invasive ventilation: critical analysis and future perspectives. Intensive Care Med. 2011;38:160–7.
