# Supplementary material for: Combined effects of leaks, respiratory system properties and upper airway patency on the performance of home ventilators: a bench study
Source: BMC Pulm Med. 2017 Nov 21;17:145. doi: 10.1186/s12890-017-0487-2 (PMC5697337; doi:10.1186/s12890-017-0487-2)
Supplement: Supplementary file 2 — Study of equivalent ventilatory settings. Following ventilatory settings were analyzed: inspiratory trigger sensitivity, pressure rise time and I:E cycling. (PDF 265 kb) [file 12890_2017_487_MOESM2_ESM.pdf]

Combined effects of leaks, respiratory system properties and upper airway patency on the performance of home ventilators: A bench study

Kaixian Zhu, Claudio Rabec, Jésus Gonzalez-Bermejo, Sébastien Hardy, Sami Aouf, Pierre Escourrou and Gabriel Roisman

Additional File 2: Study of equivalent ventilatory settings

The three included ventilators were firstly evaluated for their ventilatory settings, such as inspiratory trigger sensitivity, pressure rise time and I:E cycling, in order to establish equivalent settings for the study.

The ventilator was connected to ASL5000 through a 1.8-meter long and 22-mm diameter tubing. A calibrated intentional leak port (24 L/min at 10 cmH_2O) was coupled at the outlet of ASL5000. The ASL5000 was configured to simulate an obstructive respiratory system (COPD condition) with the resistance and compliance set at 20 $cmH_2O \cdot s \cdot L^{-1}$ and 50 $ml \cdot cmH_2O^{-1}$ respectively.

i. Inspiratory trigger sensitivity

The ASL5000 generated breathing efforts with inspiratory muscular pressure (Pmus) varying from 1 cmH_2O to 10 cmH_2O in a stepwise manner with increment of 1 cmH_2O. Each effort was repeated 15 times (20 times for Pmus=1 cmH_2O). The ventilators were set to spontaneous pressure mode which delivered spontaneous-triggered ventilatory supports. The other ventilatory settings are shown in Table S2-1.

Table S2-1: Ventilatory settings of the ventilators for the study of inspiratory trigger sensitivity

	Mode	Rising time	I :E cycling	Ti min-max (s)
A150	S	200 ms	Medium	0,8-1,5
T100	S	2	40%	1,5
V60	Support	2	4	0,8-1,5

Bpm: breath per minute. A150: Astral^{TM} 150; T100: Trilogy^{TM} 100; V60: Vivo^{TM} 60. Since the breathing rate of V60 cannot be disabled, a minimum breathing rate that equal to 4 bpm was applied.

For each inspiratory trigger sensitivity setting, the minimal Pmus which could trigger the ventilator without autotriggered cycles was noted. An example is given in Figure S2-1.

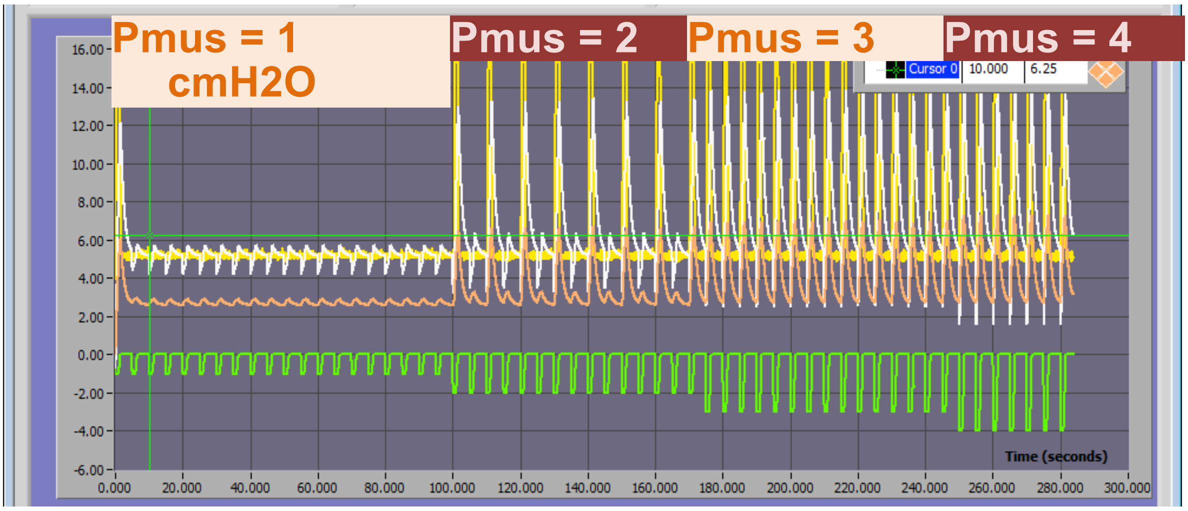

Figure S2-1: Example of inspiratory trigger sensitivity setting test. Yellow curve: airway pressure; Green curve: Pmus. The minimal Pmus which can trigger the device without autotriggered cycles was noted. In this example, the inspiratory trigger of A150 was set at medium and the minimal Pmus was 3 cmH_2O.

Thus, the equivalent inspiratory trigger sensitivity settings among the three ventilators could be obtained and the results are shown in Table S2-2.

Table S2-2: Comparison of inspiratory trigger settings between ventilators.

Pmus (cmH_2O)	Corresponding inspiratory peak flow (L/min)	Inspiratory trigger sensitivity settings with which the ventilator responds without autotriggered cycles			
		**A150**	**T100 (L/min*)**	**V60 (a.u.)**	
1	2.5		1, 2	1**	Most sensitive
2	5	Very high and High	3, 4, 5	2, 3, 4, 5	
3	7.5	Medium	6, 7	6, 7, 8, 9	
4	10	Low	8, 9		
5	12.5				
6	15	Very low			Most insensitive

* according to the user manual of T100; **Autotriggered cycles were already present. Pmus: inspiratory muscular pressure. A150: Astral™ 150; T100: Trilogy™ 100; V60: Vivo™ 60. Note that the inspiratory trigger sensitivity settings of V60 are presented as arbitrary unit (a.u.).

According to our protocol which is mentioned in the main text, each test was started with the intermediate inspiratory trigger sensitivity for each ventilator, i.e., "Medium" for A150, "5" for T100 and V60.

ii. Pressure rise time

The ASL5000 was configured in passive lung mode which did not present any breathing effort. The ventilators were set to pressure-control ventilation mode delivering mandatory ventilatory cycles at a rate of 12 breath per minute (bpm). The other ventilatory settings are shown in Table S2-3.

Table S2-3: Ventilatory settings of the ventilators for the study of rising time

	Mode	EPAP-IPAP (cmH_2O)	Inspiratory trigger	Ti (s)	Breathing rate (bpm)
A150	PAC	5-15	OFF	1.5	12
T100	T	5-15	n/a	1.5	12
V60	Assist/Control	5-15	OFF	1.5	12

EPAP: expiratory positive airway pressure; IPAP: inspiratory positive airway pressure. Ti: inspiratory time. A150: Astral™ 150; T100: Trilogy™ 100; V60: Vivo™ 60.

The time required for airway pressure to rise from 10% to 90% of the demanded pressure support (6 to 14 cmH_2O) were measured and averaged over 5 cycles. An example is shown in Figure S2-2.

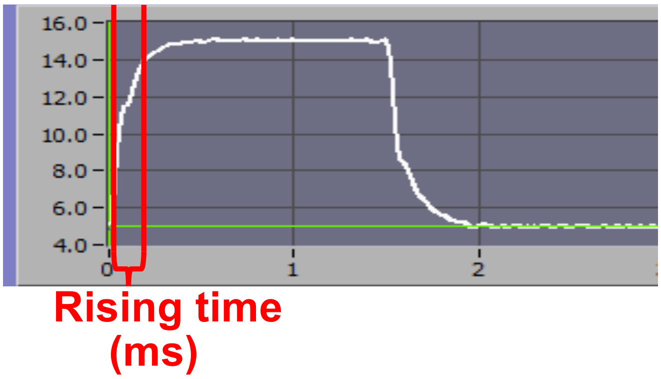

Figure S2-2: Example of rising time setting test. The time required for the airway pressure to rise from 10% to 90% (6 to 14 cmH_2O in this example) was measured.

Results are shown in Table S2-4. The rising time settings chosen for the three home ventilators in the current study are marked in bold characters in Table S2-4.

Table S2-4. Comparison of pressure rising time settings between ventilators.

A150		T100		V60	
Settings (ms)	Measured time (ms)	Settings	Measured time (ms)	Settings	Measured time (ms)
Min	136	**1**	**197**	1	142
150	175	2	410	**2**	**229**
**200**	**215**	3	584	3	306
300	297	4	800	4	370
400	382	5	995	5	455
500	479	6	1192	6	516
600	568			7	581
				8	647
				9	716

Results were presented as mean values averaged over 5 ventilatory cycles. A150: Astral™ 150; T100: Trilogy™ 100; V60: Vivo™ 60.

iii. Cycling from I to E (in %)

The ASL5000 generated breathing efforts with Pmus equal to 4 cmH_2O to trigger the ventilatory cycles. The ventilators were set to spontaneous pressure mode. The other ventilatory settings are shown in Table S2-5.

Table S2-5: Ventilatory settings of the ventilators for the study of I:E cycling

	Mode	EPAP-IPAP	Inspiratory trigger	Rising time	Ti min-max (s)	Breathing rate. (bpm)
A150	S	5-15	Medium	Min	0.1-3	OFF
T100	S	5-15	6	1	3	n/a
V60	Support	5-15	6	1	0.3-3	4

EPAP: expiratory positive airway pressure; IPAP: inspiratory positive airway pressure. Ti: inspiratory time. A150: Astral™ 150; T100: Trilogy™ 100; V60: Vivo™ 60.

The ratio of flow amplitude between the value at the end of inspiratory support (switching from IPAP to EPAP) and the maximal value were calculated and averaged over 5 cycles. An example is given in Figure S2-3.

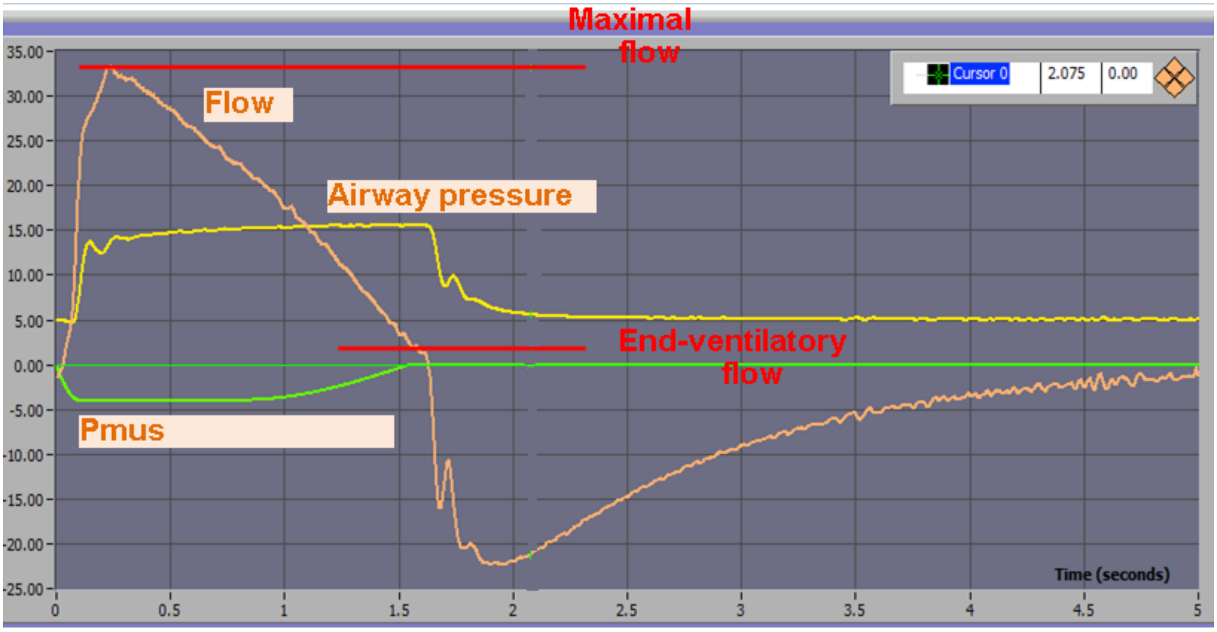

Figure S2-3: Example of I:E cycling setting test. The ratio of flow amplitude between the value at the end of inspiratory support and the maximal value were calculated. In this example, the I:E cycling of V60 was set at 9, and the obtained ratio was equal to 4%.

Results are shown in Table S2-6. The I:E cycling settings chosen for the three home ventilators in the current study are marked in bold characters in Table S2-6.

Table S2-6. Comparison of I:E cycling settings between ventilators

A150		T100		V60	
Settings	Ratio between end insp. support and maxi flow (%)	Settings	Ratio between end insp. support and maxi flow (%)	Settings	Ratio between end insp. support and maxi flow (%)
Very low	7	10	11	1	85
Low	12	20	20	2	74
Medium	26	30	28	3	63
**High**	**35**	**40**	**38**	4	51
Very high	50	50	46	**5**	**41**
		60	60	6	32
		70	68	7	23
		80	77	8	13
		90	87	9	4

Results were presented as mean values averaged over 5 ventilatory cycles. A150: Astral™ 150; T100: Trilogy™ 100; V60: Vivo™ 60.
