# Supplementary material for: Combined effects of leaks, respiratory system properties and upper airway patency on the performance of home ventilators: a bench study
Source: BMC Pulm Med. 2017 Nov 21;17:145. doi: 10.1186/s12890-017-0487-2 (PMC5697337; doi:10.1186/s12890-017-0487-2)
Supplement: Supplementary file 3 — Comparison of leaks between device reports and bench-measured values. Protocol and results of comparing the leak values between device reports and bench measures. (PDF 232 kb) [file 12890_2017_487_MOESM3_ESM.pdf]

Combined effects of leaks, respiratory system properties and upper airway patency on the performance of home ventilators: A bench study

Kaixian Zhu, Claudio Rabec, Jésus Gonzalez-Bermejo, Sébastien Hardy, Sami Aouf, Pierre Escourrou and Gabriel Roisman

Additional File 3: Comparison of leaks between device reports and bench-measured values

i. Protocol

To compare the accuracy of estimated leaks provided by the devices, each device was connected to the bench model configured as COPD condition (Additional File 1) with open UAs. Tests were performed with intermediate inspiratory trigger sensitivity for each device and other settings were identical to that shown in Table 1 in the main text. Each ventilator was successively tested with three calibrated total leak levels (24, 48 and 72 L/min at 10 cmH_2O) for 5 minutes. The mean total leaks (median for A150) were compared between the device reports and the bench-measured values. Of note, A150 reports mean leak value during cycles and intentional leak is not included (1). For T100, the device-provided leak is the average leak of the whole breathing cycle and includes intentional and non-intentional leaks (2–4). Differently, V60 provides average leak during EPAP (i.e., the lowest value during the cycle) and includes intentional and non-intentional leaks (2,3,5).

ii. Results

The Bland-Altman plots showing the differences between the total leak values obtained from the device reports and that measured on the bench are shown in Figure S3-1. The means differences were -17.5±0.8, 3.1±0.8 and -5.5±3.8 L/min respectively for A150, T100 and V60. For V60, the higher the total leak, the greater the bias compared to the bench-measured value: the leak in the device report underestimated the measured leak of 56 L/min by 9.6 L/min (Figure S3-1).

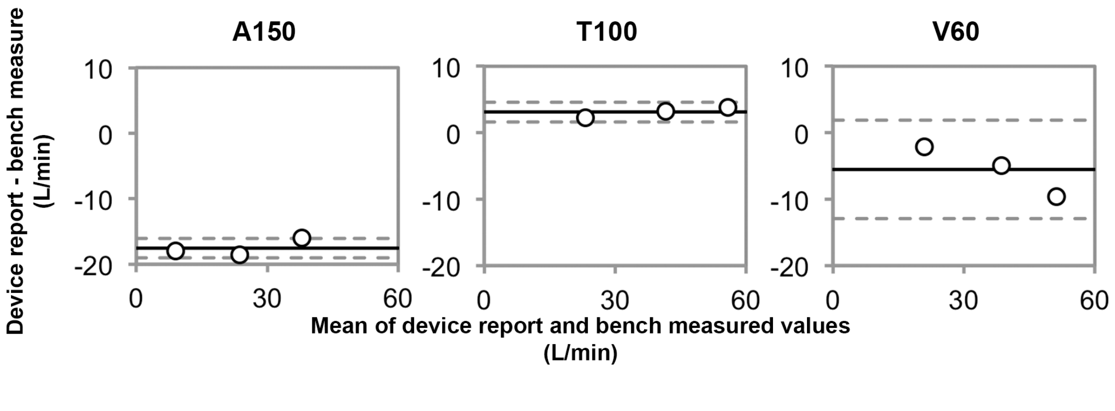

Figure S3-1: Bland-Altman plots: device-reported total leak values *vs.* bench measured values. Median leak values are presented for A150 and mean leak values are presented for T100 and V60. The solid lines represent the bias (average differences between device reports data and bench-measured values), and the dashed

lines represent the limits of agreement at 95% CI for these differences (± 1.96 SD). A150: Astral 150; T100: Trilogy 100; V60: Vivo 60. Note that A150 reported non-intentional leak while other two devices reported total leak.

----

1. ResMed Ltd. Astral series user guide. 2014.
2. Contal O, Vignaux L, Combescure C, Pepin J-L, Jolliet P, Janssens J-P. Monitoring of noninvasive ventilation by built-in software of home bilevel ventilators: A bench study. Chest. 2012 Feb 1;141(2):469–76.
3. Pasquina P, Adler D, Farr P, Bourqui P, Bridevaux PO, Janssens J-P. What Does Built-In Software of Home Ventilators Tell Us? An Observational Study of 150 Patients on Home Ventilation. Respiration. 2012;83(4):293–9.
4. Respironics. Trilogy 100 clinical manual. 2008.
5. Breas Medical AB. Vivo 60 user manual.
