# Supplementary material for: Combined effects of leaks, respiratory system properties and upper airway patency on the performance of home ventilators: a bench study
Source: BMC Pulm Med. 2017 Nov 21;17:145. doi: 10.1186/s12890-017-0487-2 (PMC5697337; doi:10.1186/s12890-017-0487-2)
Supplement: Supplementary file 4 — Inspiratory trigger settings corresponding to “critical leak” values. Table of the inspiratory trigger settings of the three ventilators corresponding to the “critical leaks” according to the protocol in Fig. 3. (PDF 80 kb) [file 12890_2017_487_MOESM4_ESM.pdf]

Combined effects of leaks, respiratory system properties and upper airway patency on the performance of home ventilators: A bench study

Kaixian Zhu, Claudio Rabec, Jésus Gonzalez-Bermejo, Sébastien Hardy, Sami Aouf, Pierre Escourrou and Gabriel Roisman

Additional File 4: Inspiratory trigger settings corresponding to "critical leak" values

	COPD open UA	COPD closed UA	OHS open UA	OHS closed UA	NMD open UA	NMD closed UA
**A150**	Medium	Very high	Medium	Very high	Medium	N/A
**T100**	5	4	5	4	5	3
**V60**	5	6	9	1	5	N/A

UA: upper airway; A150: Astral™ 150; T100: Trilogy™ 100; V60: Vivo™ 60. According to the definition, the critical leak of Astral 150 and Vivo 60 could not be determined when subjected to NMD with closed UA, since these two devices could not reach an AL ≤ 25% when the minimal leak (intentional leak) was applied despite adjustments of the sensitivity of inspiratory trigger. The relationships of the inspiratory trigger settings between the devices are shown in Table S2-2, Additional File 2.
