# Supplementary material for: Combined effects of leaks, respiratory system properties and upper airway patency on the performance of home ventilators: a bench study
Source: BMC Pulm Med. 2017 Nov 21;17:145. doi: 10.1186/s12890-017-0487-2 (PMC5697337; doi:10.1186/s12890-017-0487-2)
Supplement: Supplementary file 5 — Example of patient-ventilator asynchronies with closed upper airways. Detailed curves of airway flow, pressures and leaks illustrating observed patient-ventilator asynchronies. (PDF 5414 kb) [file 12890_2017_487_MOESM5_ESM.pdf]

Combined effects of leaks, respiratory system properties and upper airway patency on the performance of home ventilators: A bench study

Kaixian Zhu, Claudio Rabec, Jésus Gonzalez-Bermejo, Sébastien Hardy, Sami Aouf, Pierre Escourrou and Gabriel Roisman

Additional File 5: Example of patient-ventilator asynchronies with closed upper airways

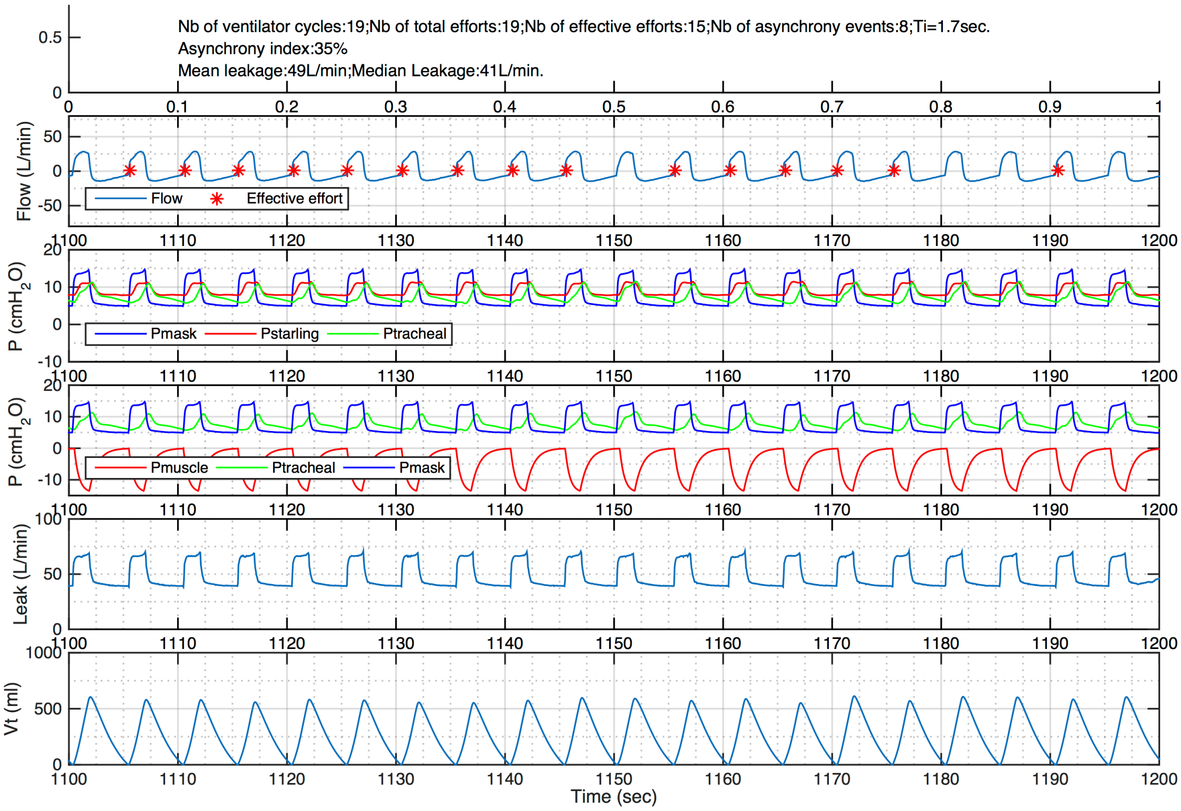

Figure S5-V1

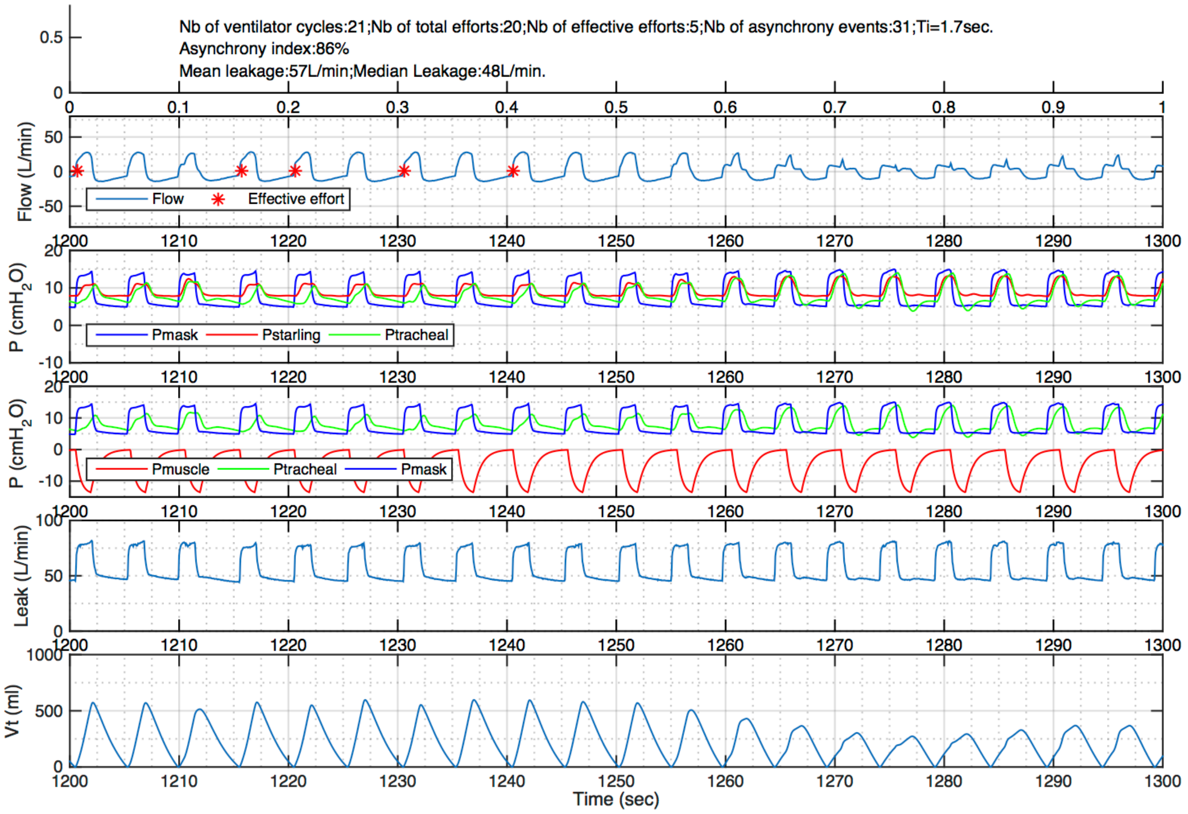

Figure S5-V2

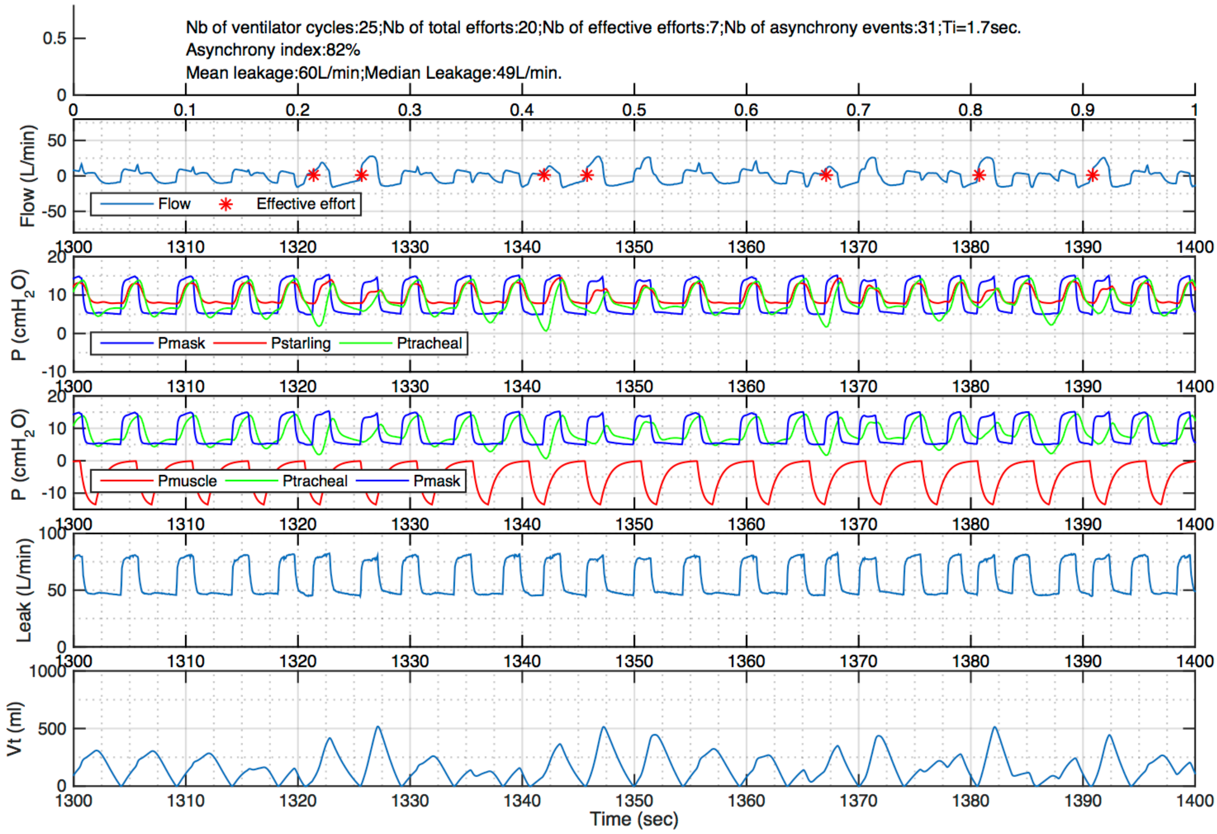

Figure S5-V3

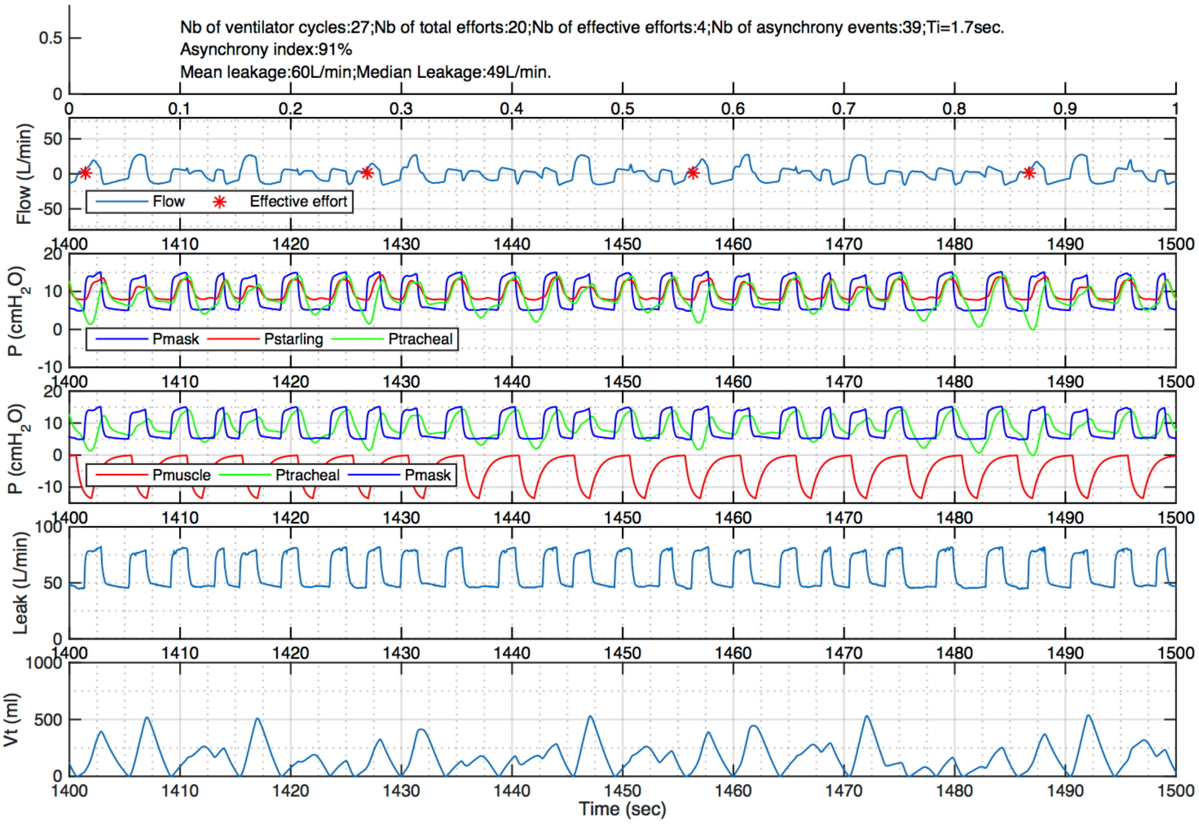

Figure S5-V4

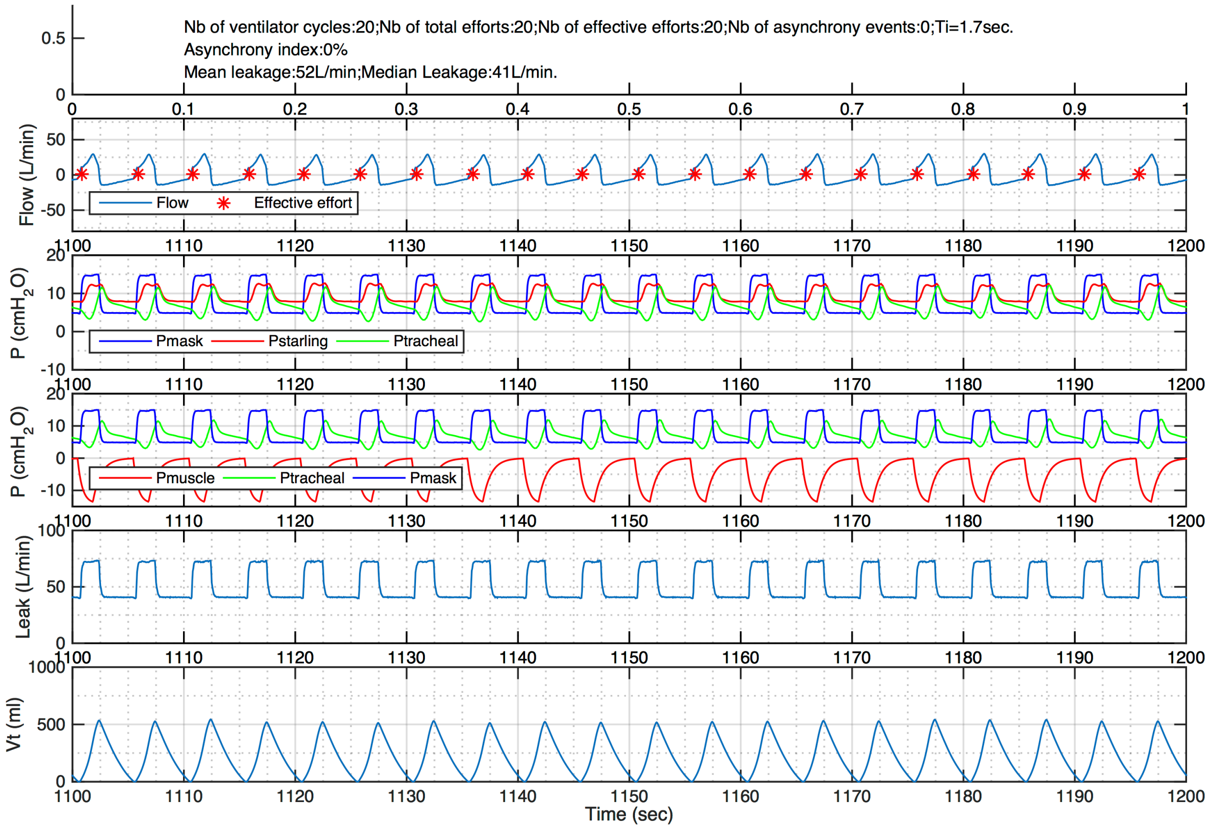

Figure S5-T1

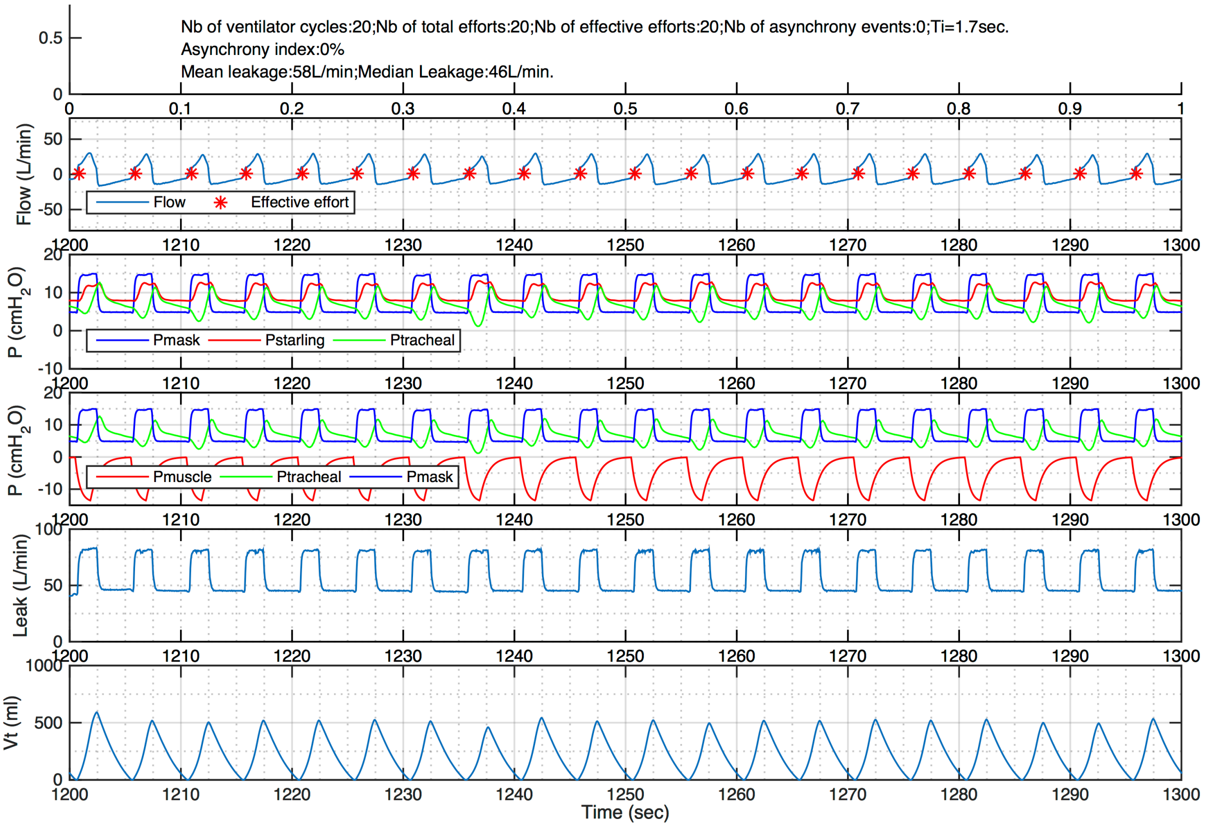

Figure S5-T2

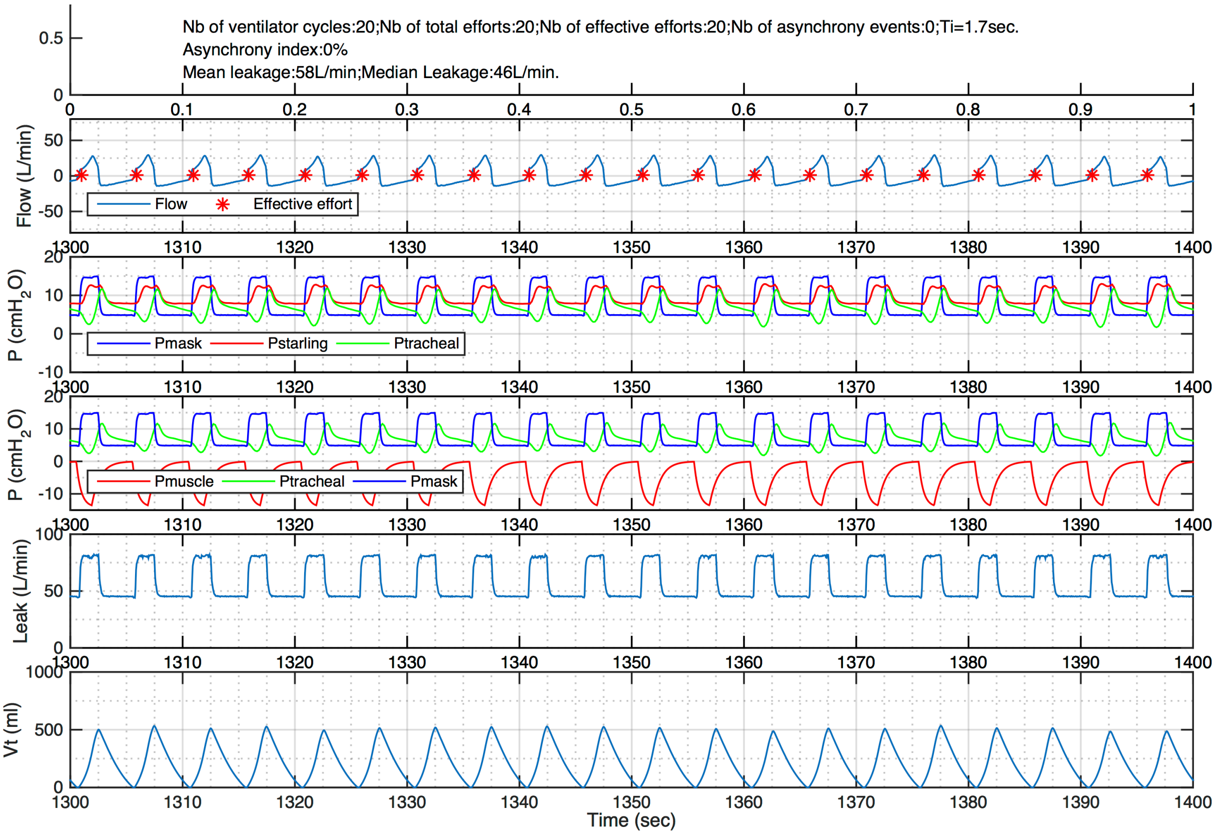

Figure S5-T3

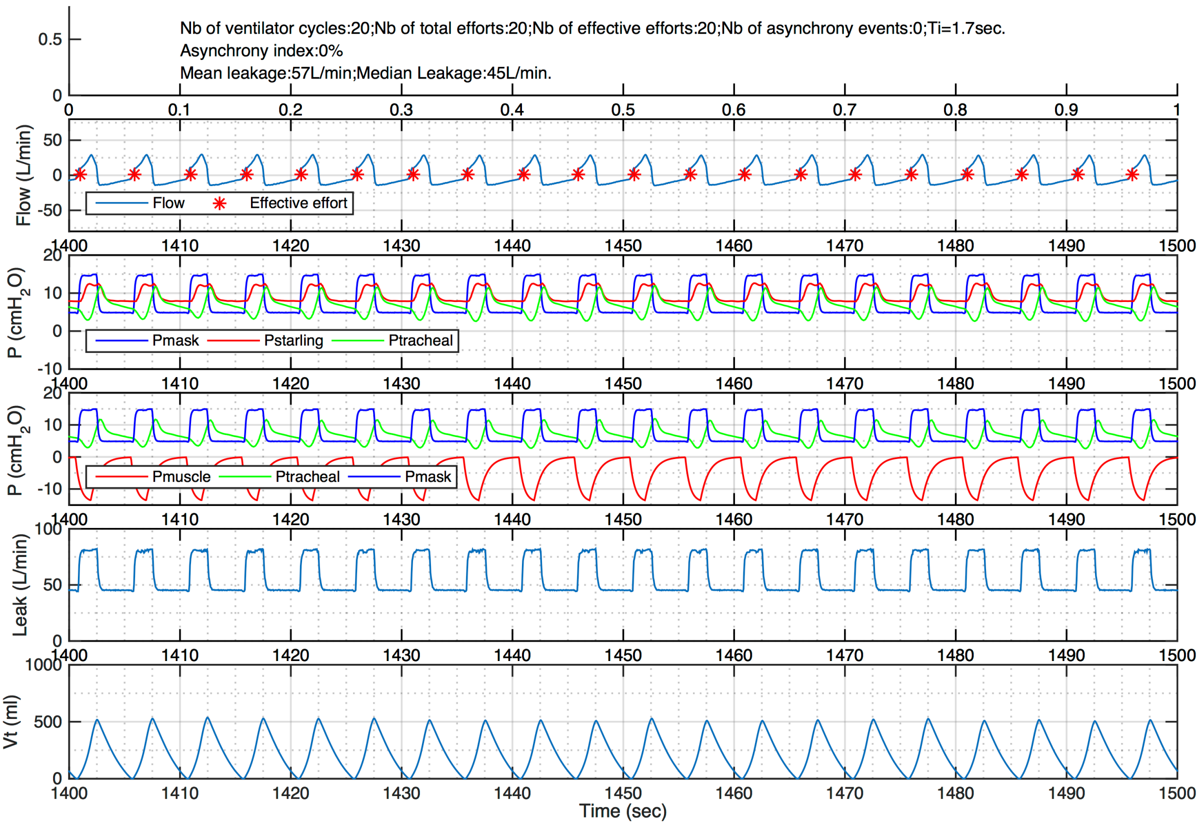

Figure S5-T4

Figures S5 V1-4 and T1-4 show mask flow, mask pressure (Pm), tracheal pressure (Ptr), inspiratory muscular pressure (Pmus), total leak and calculated tidal volume (V_T) during period between 1100th to 1500th second during the tests of V60 (V1-4) and T100 (T1-4) under COPD condition with closed upper airways. During the period shown in the figures, the two devices were subjected to similar total leak levels: from 1100th to 1200th second, median leak was around 41 L/min; at 1200th second, leak was manually increased to around 48 L/min. The evolutions of patient-ventilator interaction during this increase of leak are thus shown in the figures.

For V60, the inspiratory trigger was 6. When leak level was around 41 L/min, 15 out of 19 inspiratory efforts were effectively addressed by pressure supports sent by the ventilator, and the amount of asynchrony events was not significant. A similar level of Ptr to Pm indicated that the upper airways remained at least partially opened. The V_T reached around 600 ml. When the leak increased to 49 L/min at 1200th second, the ventilator cycles began to desynchronize to the simulated patient's breathing efforts (decreases in Pmus). Ineffective inspiratory efforts appeared leading to asynchrony and a consequent decrease in delivered V_T (from the 1255th second). Later, high frequency ventilatory cycles were auto-triggered, leading to a decrease in delivered mean V_T (from 1320th second, minimum value around 250 ml). As the breathing rate of active lung model was unchanged, an auto-triggered ventilatory cycle could occur by chance at a similar or a same moment when the inspiratory effort took

place. Consequently, an increase in V_T could be observed which was, however, completely accidental (e.g., at 1327th second, 1347th second, etc.).

The corresponding curves of T100 under similar conditions are comparatively shown in the figure. The inspiratory trigger of T100 was 4. No asynchrony events occurred.
